# Collectanea Medica: Consisting of Anecdotes, Facts, Extracts, Illustrations, &c.

**Published:** 1821-03

**Authors:** 


### COLLECTANEA MEDICA:

CONSISTING OF

### ANECDOTES, FACTS, EXTRACTS, ILLUSTRATIONS, &c.

*Relating to the History or the Art of Medicine, and the Auxiliary Sciences.*

---

Floriferis ut apes in saltibus omnia libant,

Omnia nos itidem depascimur aurea dicta.

---

### *Extracts of the Report from the Select Committee of the House of Commons on the Doctrine of the Contagion of the Plague, in* 1819.

*Veneris*, 19° *die Martij*, 1819.

SIR JOHN JACKSON, Baronet, in the Chair.

Dr. AUGUSTUS BOZZI GRANVILLE, called in; and Examined.

ARE you acquainted with the plague, and have you formed any opinion as to its being contagious: favour the Committee with your opinion respecting it?—I have seen the plague, and I have no doubt that it can be conveyed by an individual infected by it, to another in perfect health.

Where have you seen the plague?—In various parts of Turkey, Greece, Asia, Syria, Egypt, &c. and in Constantinople, where I resided two years.

What are the symptoms and characteristics of plague?—The symptoms are permanent; and, during the time the plague raged in Turkey in 1812, they were permanent throughout the country. It is a sudden dizziness, great pain in the head, great prostration of strength, affections of the nervous system particularly; (there are no symptoms of inflammation whatever, not such as attend inflammatory diseases during their first attack;) sickness of the stomach occasionally, and the invariable appearance of glandular swellings, if it goes beyond sixty hours.

Is it not also attended with carbuncles?—With carbuncles and other pestilential eruptions, particularly livid spots on the body, partial mortification of the body.

What do you consider the cause of the plague in Turkey?—It is a question no practical man can answer; it is entirely unveiling the mystery in which all diseases are enveloped: I can only answer that it does exist, and is conveyed in the way I have stated.

To what do you ascribe our not having it in Great Britain?—To the regulations of the quarantine laws. It appears to me, as far as I can judge of the nature of a disease without knowing its origin, that, being endemical at certain parts of the globe, it might explain why it is not peculiar to this country unless imported.

Does it not often appear in places in the Mediterranean, where the quarantine-laws are severely rigid?—Never, except in lazarettos, or where a violation of those laws has taken place.

Then you conceive it must be imported, and that it cannot originate in those places?—It does not originate; certainly not.

What precautions are taken to prevent infection, by the Frank inhabitants of Smyrna and Constantinople, and other places visited by the plague?—If they can afford it, shutting themselves up in the houses before communication with persons infected; if they are obliged to go abroad, as some are, such as physicians who have their livelihood to get, some wear oil-skin dresses, oil-skin gloves, and other medical precautions to prevent breathing the infected air; others anoint themselves with oil, and avoid contact as much as possible, under a strong persuasion that contact produces disease. In Egypt and Syria they shut themselves up as soon as there is a rumour of the plague, and never quit till the dews fall, that is, till St. John's day; then they come out, and proceed to church in order to sing Te Deum.

During the prevalence of great disease in any of these towns, did you ever know the plague destructive in the families of the Franks who avoid contact with diseased persons?—No one instance, where contact or some other conveyance, by goods or other articles, could not be ascertained. If the Committee will allow, I will state a circumstance as to the house in which I lived myself. The house was one of the chief houses of the hospadar of Wallachia, Prince Suzzo; that was the house where I lived for eleven months, during which time, at one period, in 1803, the plague prevailed; the precaution of shutting up the family immediately on the appearance of the plague, was never omitted; and no one case was on record in the family, of any plague having occurred within the walls of it.

Could such a circumstance possibly have occurred, provided the plague had been an epidemic disorder: could the shutting-up of one house in an infected city save the family?—Certainly not. But the plague is not epidemic. I can bring cases in support of the assertion with regard to the plague not being an epidemic distemper, and that it does not depend on atmosphere or ventilation.

Do you not conceive that, in the case of contagion, the state of the air may have the strongest effect in stopping or promoting the effects of the disorder, as you have instanced in the case of the procession on St. John's day?—It may render the person exposed to the contact more or less liable to feel its effect, but will not operate in checking the disease.

To what do you attribute the periodical appearance of the plague in the spring and autumn?—Because the seasons have an influence on the character of the disorder, the same as in this country: in winter you are more likely to catch a cold or catarrh.

Can you guess how plague was originally produced?—I should premise by stating that the impossibility of ascertaining the origin of a disease does not do away with its existence. Every medical man has attempted to form an opinion; and, I should state in answer, it is most probably an epidemic disease at some particular parts of Egypt. The first mention of it is as coming from that country.

What authors do you refer to?—First to Thucydides; though I am myself of opinion, that the plague of Athens, mentioned by him, was not the plague of the present day. The other authors are Muratori, Guastaldi, Foderé, Nacquart, and very recently Jourdan and Valli.

Are cases of plague pretty frequent in the division of Constantinople called Pera?—Not so frequent, of course, as in Constantinople, because every Frank takes precaution against the disease.

Is the suburb of Pera differently built from Constantinople?—It is a little more elevated, and is a long narrow street. As to the houses, many of them are of stone, whereas in Constantinople they are chiefly wood; and the streets are wider at Pera than they are at Constantinople, generally speaking.

Is Pera upon the whole, from its situation, a more airy place than Constantinople?—More than some parts.

Do you think Pera is a less likely situation for the production of any disorder peculiar to the climate, than Constantinople?—I should say that there is no difference, except with regard to the crowded state of the houses; the topographical difference would not make much. The street leading from Constantinople to Pera is one of the dirtiest streets in Constantinople.

Have you been at Aleppo?—I have been at Aleppo.

Do you know that caravans proceed very frequently, for the conveyance of goods from Aleppo eastward, through the continent of Asia?—Certainly.

Have you ever heard that the plague was conveyed by those caravans eastward, so as to establish itself?—Not except some of the few thinking Christians; the mass of the people never think of the disease at all. I have heard it among a few persons I have conversed with, and who thought the plague could be carried.

Has it actually been carried eastward?—I have no knowledge myself.

Do you believe it?—I do believe it.

Have you heard so?—I have heard so, but I do not know it.

In cloth carried eastward?—In the caravans leaving Aleppo. I have no knowledge myself of the fact.

Have you ever heard of any city to which the caravans proceeded, becoming the seat of the plague?—Damascus, in 1804; it was carried by the army of some pashwa, who had been on the coast to assist in the reduction of Jean d'Acre. Bagdad is often, and has been lately, infected with the plague.

Have you heard of any other instance?—None from Aleppo.

And none of the plague carried eastward?—Not to my own knowledge.

You have not heard of the plague establishing itself, and destroying the inhabitants, in any of the interior cities of Syria, except in Damascus in 1804?—I have had no means of investigating that.

Have you ever been in Smyrna?—I have been in Smyrna.

At the time of the plague?—Not when it was very prevalent; but when a few cases were reported to the different consuls, so as to induce them to give to the vessels a bill of health, which is called a Suspicious Bill, *touched*; that is, infected.

Have you ever heard of the plague being communicated from Smyrna to the interior cities of Asia Minor?—I have not heard any particulars.

Have you ever heard of the plague being at Brusa in Bythinia, eastward of Constantinople?—I will not take on myself to say.

Have you heard of the plague being communicated westward of Constantinople, over land, to Adrianople and other cities?—I have.

To Adrianople?—Yes.

Do you know instances of the plague being destructive at Adrianople?—I believe the plague raging in 1812 was nearly as fatal as it proved at Constantinople.

Have you heard of the plague being communicated from vessels from Smyrna to many parts of the Levant?—Continually.

What is your opinion as to the infectious nature of the plague to be carried in bales of merchandize?—If the word infectious is to stand in my examination, I beg leave to qualify it. The plague is not infectious, as the yellow fever is; but it is contagious, which explains the way in which it is carried; for infection cannot be carried.

Explain the difference between infection and contagion?—Contagion is a mere mode of action resulting from the habit of certain diseases to affect individuals; it is not a principle, such as the electric fluid and such kind, as many persons give an idea of in their writings, flying about the air. Contagion expresses this: during such a disease as the plague, there are certain animal emanations which partake of the morbid state of the body from which they issue; when these are applied by direct contact, or by any mediate contact, namely, objects on which these emanations rested, to an healthy body, it will contract the disease. Infection is this: infection is a peculiar state of the atmosphere, which has been rendered unfit for the healthy exercise of life, by the crowding together of a number of persons ill of the same fever in a given place, and during a given time; thus an epidemic may become infectious.

You mean epidemic influence. What is your opinion of the contagious nature of the plague to be carried in merchandize from one country to another?—The answer is included in the one precedently given: I stated that the plague, and two or three other contagious diseases, seem to give out, as the body does, certain emanations, which must partake of the same disease as the body. If these are applied to goods liable to receive and nurse it, even then the principle of the disease, (if you call it the principle, but I am averse to such a name,) may be conveyed by such articles containing the emanations being carried about.

Do you think that articles so contaminated by the matter of the plague would retain the matter for a long time, so as to communicate the disorder upon being touched?—There are examples, and those very authentic, proving that this matter of the plague can, if applied to an healthy body, cause the disease to break out even at a very long period after; and I should mention, several months. There is one instance in point among the most recent, and it rests on the highest authority. During the plague at Corfu in 1815, one of the villages, which had been infected several months, had for some time, I believe for forty-three days, exhibited no sign of the plague, owing to the measures of segregation adopted by Sir Thomas Maitland; the village was reported to be released, and fumigation, preparatory to its receiving *pratique*, ordered. The officer who had the surveillance of the village, during the three or four months had resided in the church, from there being no house that was not thought infected; in which church the people and the priest had been crowded just before the laws of segregation were ordered by Sir Thomas Maitland: some of these died subsequently, for the church was ordered to be shut the instant the plague begun. It was therefore necessary to purify the church before the people could go in again, as well as the village altogether. Leave being granted, the priest went in, and touched the cloth of the great altar, so as to shake it to purify it, when he was seized with the plague, beginning with the head-ach, so as to cause him to fall on the steps of the altar almost immediately; and in three hours, before he could be carried to the lazaretto, he expired, with buboes under the arm and livid spots over the body.

I thought you said, in a former part of your evidence, that the buboes only appeared when the disorder continued certain hours?—When it continues sixty hours, they are sure of coming, but they may be earlier. I merely said that the glandular swellings must appear if the disorder goes beyond sixty hours.

Do you think, from your general knowledge of physiology, that the priest then took for the first time the infection of the plague, and sickened on the spot, so as to die of the plague in three hours from the time of touching the cloth of the altar?—From analogy to the rapid action of other poisonous substances with which I am acquainted, I should think there was not the least doubt as to the priest having been infected for the first time.

And do you think he took the disorder by contact through the skin of his hand with which he touched the cloth, or by any effluvia from the cloth which he might have inhaled at the time, when he went so near as to touch it with his hand?—Certainly through the skin; because, if the effluvia had arisen from the cloth, there is no reason why the officers who had resided there two months shut up in the church, should not have felt the effect.

Did he fall down upon the steps of the altar, immediately after touching the cloth?—He was seized with a dizziness of the head; touched with the plague: there came on dizziness, and he fell in a fainting-fit; he was seized with the plague. It is the usual expression. The case is stated thus: he was seized with the plague, and died in three hours afterwards.

Do you believe the case to be exactly as you have reported it?—From my own knowledge of several facts, such as I can have no hesitation in believing.

Have you seen such instances yourself?—I have not seen them myself; but I have had them from such good authorities, where I met with them, that I cannot doubt their truth.

And such an event is agreeable to your general knowledge of physiology?—Certainly; I do not see any law in physiology that can prevent the belief that virulent poisons can be carried into circulation, and go through the lymphatic system in less time.

Then, according to your opinion, the plague must be brought: the contagious matter of the plague must be brought either by persons or in bales of goods on-board ship from the Levant to England; and persons touching the infected portion of merchandize packed in these bales, must exhibit such phenomena in the lazarettos in England as you have described to have been reported to you to have happened in the village in Corfu?—Without the smallest doubt, that is my firm belief. Cases in point have happened at the lazaretto at Leghorn, since 1814; at Marseilles within fifteen years, twice; and recently, according to the dispatches of Mr. Hoppner, the British consul at Venice, in October 1818.

But you have not heard of such instances in the lazaretto in England?—No.

Have you ever heard of the plague being caught by any of those persons appointed to see the quarantine-laws put in execution in the lazarettos in England, and who in discharge of their duty must be liable to such communication with goods and persons as would expose them, one would think, to the contagion?—I have not heard of any cases of the kind happening in England, to my personal knowledge; but I have never made inquiry in England.

Do you think it extraordinary that, in the lazarettos in England, none of the officers appointed to carry the quarantine-laws into execution should not have caught the plague?—In order to answer whether it is extraordinary or not, I ought to know whether a vessel arriving at a lazaretto has performed any quarantine or made any stay in the Mediterranean ports; whether they have come direct; whether, before the bales have been touched, there has been purification and other means of precaution observed.

You have stated, that the contagious matter of the plague, being entangled in goods fit to preserve it, may remain in a state to communicate the disorder for many months?—I do think so.

Do you not think it likely, that by some accident in a long series of years, from the contagious matter of the plague, and from the nature you think it to be, it might have been introduced into England from the ports of the Levant?—I should humbly conceive, that its non-appearance in England does not do away with the contagious nature of the disease: it must be supposed that the goods had the plague; I should say, then, that the expurgators could not avoid having the plague.

Then, as they never had the plague in our lazarettos, you naturally conclude that those goods which arrived were not, on their arrival, infected with the plague?—Certainly not, if they had been touched by several individuals on their arrival before purification, and they had not excited symptoms.

Are you aware that, during the prevalence of the plague in the Levant, goods are in general not allowed to be shipped for England, under the quarantine-laws, till after the disease has ceased?—I am perfectly aware of it.

Would not the length of time after such goods were shipped, and the length of the road to England being so great, be sufficient to account for the contagion being considerably weakened ?—If the length of time is very great between the time of shipping and unloading, and if certain circumstances have taken place, either on the removal of the cargo during the voyage, or in altering it, or the vessel's meeting with bad weather and being washed over and over again, it is not improbable to suppose that part of the plague-matter, if any existed in the cargo or attached to any part of the vessel, may have been weakened in its virulence: but I beg to give that as a supposition, and not as my belief, because we know that all poisons may be qualified by many circumstances, so that the strongest may not have effect. A barrel of gunpowder may not take fire with a red-hot poker, under certain circumstances; that is, if by moisture you render it incapable of combustion.

Are not different persons, in different climates, more or less susceptible in various degrees of contagion ?—No doubt: the prevalence of certain circumstances, both with respect to individuals and climates or seasons, would very much forward or diminish the chance of the person receiving the effect of contact.

Would not these circumstances, and the length of time which elapses in a voyage between the Levant and England, account for the very rare degree of contagion which has taken place in England, without supposing the impossibility of it?—I should think it scarcely probable, that if what I have called a contagious matter is in the bales of goods, unless the period of time is very great, that it would fail to excite the disease.

Would that account for its not having taken place during the last 154 years?—The only way I can account for its not having taken place is, that it was never shipped from the Levant; for, though I admit there is a probability that circumstances will diminish the virulence, I do not admit that, if the disease is shipped on-board, any circumstances will prevent its spreading.

Is not the fact of its never having occurred for 154 years sufficient to inspire the confidence that it cannot exist in a British atmosphere? —Neither 154 years, nor six or seven centuries, can give such hope, when we know that such a disease existed before.

Was the plague of 1665 the plague of the Levant?—No doubt, according to Dr. Mead.

